# Feasibility of Laparoscopic Appendectomy Among Children in Nepal: A Safe and Preferred Approach

**DOI:** 10.7759/cureus.90351

**Published:** 2025-08-17

**Authors:** Lok B Kathayat, Bharat M Banjade, Bikram Duwal, Jasmine Bajracharya, Ritesh Shrestha, Rabin Koirala

**Affiliations:** 1 General Surgery, James Paget University Hospital NHS Foundation Trust, Great Yarmouth, GBR; 2 Surgery, ADh. Atoll Hospital, Ministry of Health, Mahibadhoo, MDV; 3 Surgery, Nepal Cancer Hospital and Research Centre, Lalitpur, NPL; 4 Pediatric Surgery, Nepal Medical College and Teaching Hospital, Kathmandu, NPL; 5 General and GI Surgery, Helping Hands Community Hospital, Kathmandu, NPL

**Keywords:** acute appendicitis, appendectomy, laparoscopic pediatric surgery, laproscopic appendectomy, “nepal”

## Abstract

Background

Acute appendicitis is a common condition in children, and appendectomy is the treatment of choice. The laparoscopic approach has many advantages over the open approach and should be the first choice. Open appendectomy is still a common practice in Nepal. Although laparoscopic appendectomy offers advantages over open surgery, its feasibility in the pediatric population of resource-limited settings like Nepal has not been well studied. This study was conducted to determine the feasibility of laparoscopic appendectomy in terms of technical difficulty, operating time, complications, and early recovery without further adding the risks of general anaesthesia.

Methods

This was a single-centred prospective observational study conducted at a tertiary centre hospital in Nepal for the period of one year. All children between the ages of 5 and 15 with acute appendicitis were included in the study and followed up till day 30 after discharge. Data were entered in Microsoft Excel (Microsoft Corp., Redmond, WA, USA), and the statistical calculations were carried out using Statistical Package for the Social Sciences (SPSS) version 16.0 (SPSS Inc., Chicago, IL, USA). Descriptive and inferential statistical measures were used for data analysis, and the results are represented in tables.

Results

Altogether, 17 children (53.12%) were between 11 and 15 years old, with a male predominance. Most of the patients presented early to the hospital, with a mean duration of 18 hours after the onset of symptoms. Seven children (21.88%) had an appendicolith and four children (12.50%) had a perforation at the shaft. Mean operative time was 44.9 ± 15 minutes. There were no intra-operative and immediate post-operative complications as well as the need of conversion to open. All children tolerated the general anaesthesia well. Only one (3.13%) patient had umbilical port surgical site infection (Southampton Grade Ic and Clavien-Dindo Grade I). Most of the patients were discharged on the second day, and none of them required readmission.

Conclusions

In the hands of experienced surgeons, laparoscopic appendectomy appears safe and feasible among children in a resource-limited country like Nepal. Intra-operative and post-operative complications appear minimal, allowing for early discharge with a small scar.

## Introduction

Acute appendicitis (AA) is the most common cause of the acute abdomen, thereby seeking emergency surgical care [1]. It is relatively rare in infants but very common in childhood and early adult life, reaching its peak in teens and early twenties with a slightly male predominance of 3:2 [2,3]. There is significant variation in the presentation, severity of the disease, radiological workup, and surgical management of patients with AA related to country income [4]. Some studies have evolved in the non-operative management of this condition, but appendectomy remains the treatment of choice [5]. Since perforation and peritonitis are common complications in extremes of age, appendectomy should be the option of choice in these groups [2].

The laparoscopic approach provides considerable benefits over the open approach, including a small scar, a shorter hospital stay, less postoperative pain, earlier postoperative recovery, and lower postoperative complication rates, such as surgical site infection and adhesive bowel obstruction [6,7]. Technical difficulty in handling the instruments during laparoscopic surgery may be a concerning factor in children due to their small abdominal cavities. However, studies have proven that it can be done as effectively as in adults [8,9].

Open appendectomy (OA) may be a regular practice in resource-limited or some teaching hospitals with trainee surgeons. Studies have proven the feasibility of laparoscopic appendectomy (LA) at a tertiary-level hospital in a developing country [8].

In the expert’s hand, there is only around a 15-30 minute difference in mean operating time between OA and LA (49.2 + 18.1 minutes vs. 78.2 + 25.7 minutes; p < 0.05), and almost all of the LA can be completed within 1.5 hours in maximum [10,11].

Anesthesia in children is another concern. Most parents even fear general anesthesia (GA) and surgical risks to their children. Due to this, they may request conservative treatment or an open approach with spinal anesthesia [12]. Nonetheless, anxious and uncooperative children may be problematic with spinal anesthesia, which has a significant failure rate and has not been demonstrated to be superior to general anesthesia (GA) [13]. Studies have shown GA to be safe in most healthy children, even children under 3 years, without adverse neurodevelopmental outcomes [14].

Especially in low-income countries like Nepal, where there is no insurance coverage, cost-effectiveness and the availability of laparoscopic setup play an important role. One study from Nepal strongly suggested LA to be the gold standard due to its advantages of lower incidence of wound infection, lesser postoperative analgesic requirement, and shorter hospital stay [15]. Another study in a teaching hospital in Nepal found a cost difference of NRs 6720 (~50 USD) between the LA and OA groups. This cost may be variable in private sectors and individual hospital setups. This study recommended that the patient select either one with not much added advantage on either side [16]. Similar studies in the Western world, with insurance coverage, challenged LA in pediatric patients only in terms of cost-effectiveness [17,18]. In Nepal’s context, the cost difference of the operating charge of NRs 6720 (~50 USD) should not limit a child from the advantages of LA over OA. This cost has not covered the total cost of living, including prolonged hospital stays and the cost of the utility of caregivers. Instead, this may overshoot that amount.

Even though studies have shown LA as the standard care for AA, we have no data on the feasibility and outcomes in the pediatric population in Nepal. So, this study was conducted to see if LA would be feasible in this age group in terms of technical difficulties without adding any further risks to the patient, decreasing complication rates, hospital stay, as well as overall outcomes.

## Materials and methods

This was a prospective single-centred observational study conducted at one of the tertiary centre hospitals in Nepal, for a period of one year from September 2020 to August 2021.

Primary objective was to study the feasibility of laparoscopic appendectomy among children in Nepal in terms of technical difficulties and patient safety. Secondary objectives were to identify operating time, intra-operative and post-operative outcomes in terms of complications like perforation, bleeding, thermal injury, surgical site infection (SSI), abscess formation, length of hospital stays (LOS) and readmission. The primary endpoint was to assess technical feasibility, defined by the ability to complete the laparoscopic procedure safely without conversion, with minimal intra-operative complications and acceptable recovery time. The secondary endpoint was a 30-day follow-up.

All the cases were taken in a consecutive manner if they did not fall under any of the exclusion criteria. Inclusion criteria of the study were patients diagnosed with acute appendicitis and between 5 and 15 years of age. Patients with appendicular lump, unfit for laparoscopic surgery due to any reason, and those who declined to participate in the study were excluded.

The sample size was calculated in reference to a study in Bangladesh (2014) [19]. For a large population, i.e., infinite population, n=$(z^2 pq)/d^2$. Here, n is the sample size, z is the confidence interval (CI) (1.96), p is the prevalence (2.54%), q is 1-p, and d is desirable error or precision level. Considering absolute precision (d) of 5% to 10% and relative precision (d) of 10% to 20% of proportion, the value is 20% of 2.54, that is, equal to 0.50. Finally, N = 3.84 x 2.54 x 97.46 / 0.50 = 3802. Here, N is the total number of the population (i.e., there were 30 cases last year in our hospital).

For finite population (having sample frame):

n = $n_0/(1+n_0/N)$,

n = $3802/(1+3802/30)$,

n = 29.76 (approximately 30)

Here, $n_0$ is the sample size taken from the first case.

Ethical approval from the Institutional Review Committee (reference number 32-076/077) was obtained. Detailed information regarding the study was given to the patient before recruitment, and written informed consent was taken. Confidentiality was maintained throughout the study period and thereafter until voluntarily disclosed by the patient.

Diagnosis was based on clinical features and imaging findings (Ultrasound or CT scan). Detailed parameters, as mentioned in the proforma, were taken. Written informed consent for surgery was taken from the parents. A single dose of third-generation cephalosporin (Ceftriaxone) was given intravenously during induction. The procedure was completed under general anaesthesia by standard three-port (one 10 mm umbilical port by Hasson's technique for camera, two 5 mm ports in the left iliac fossa and supra-pubic region respectively as working ports) laparoscopic approach. The appendix was taken out in the retrieval bag through the umbilical port. Peritoneal lavage was done with normal saline till the wash looked grossly clear. Intra-operative findings such as perforation, complications such as bleeding and thermal burn, and post-operative complications (SSI and abscess formation), length of hospital stay, and readmission within 30 days of discharge (for evaluation of abdominal pain, fever, vomiting, and diarrhoea) were noted. Hospital stay was calculated from the date of surgery to the date of discharge. Patients were discharged once they were able to tolerate oral intake, absence of abdominal pain, fever, vomiting and improvement in the inflammatory marker (white blood cell count and C-reactive protein).

Acute appendicitis was diagnosed as per the Paediatric Appendicitis Score (PAS) supported by ultrasonography (USG) and/or computed tomography scan (CT) as needed [3]:

PAS 0-3: No appendicitis can be discharged.

PAS 4-6: Requires further imaging/repeat evaluation.

PAS ≥ 7: Requires surgery.

Standard terms like an appendicular lump, abscess, complications like a thermal burn, significant bleeding both intra- and postoperatively, surgical site infection (SSI) including superficial, deep, and organ space infection, and postoperative intra-abdominal abscess (PIAA) were defined, referencing the available literature and guidelines [2,4,9,20-23].

Data were collected in a well-designed proforma and then entered into a Microsoft Excel sheet. All statistical calculations were done using the Statistical Package for the Social Sciences (SPSS) version 16.0 (SPSS Inc., Chicago, IL, USA). Descriptive and inferential statistical measures were used for data analysis and table results.

## Results

Among 32 children enrolled in the study, the mean age was 10.78 ± 2.36 years. More than half, 17 (53.12%), were between the ages of 11 and 15 years, while the remaining 15 (46.88%) were between the ages of 5 and 10 years. Demographic profiles are shown in Table 1.

**Table 1:** Demographic profile of children

Characteristics		Frequency (n=32)	Percentage (%)	Mean Value	Standard Deviation
Gender	Male	21	65.63	-	-
	Female	11	34.37		
Age (Years)	5-10	15	46.88	10.78	2.36
	11-15	17	53.12		
Weight (Kg)	15-30	13	40.63	31.56	7.06
	31-45	19	59.37		
Height (cm)	<120	3	9.38	34.75	9.49
	121-140	20	62.50		
	141-160	9	28.12		

The duration of the presentation was variable. Most of the children, 27 (84.38%), presented within 24 hours, and the remaining 5 (15.63%) presented after 24 hours of the onset of the symptoms. The mean duration of the presentation was 18 hours (Table 2).

**Table 2:** Presentation and findings

Characteristics		Frequency (n=32)	Percentage (%)	‘P’ Value
Duration of presentation	<24 hours	27	84.38	-
	>24 hours	5	15.63	
Appendicolith		7	21.88	-
Diameter of Appendix (mm)	6-12	28	87.50	< 0.001
	>12	4	12.50	
Appendicular Perforation	Yes	4	12.50	
	No	28	87.50	
Appendicular Lump or Abscess		0	0.00	-

The appendix was variable in size, with a mean diameter of 8.16 ± 2.26 mm. Only 7 (21.88%) had evidence of an appendicolith in USG (Table 2).

Intra-operatively, no well-formed appendicular lump or abscess was encountered; 28 (87.50%) of patients were found to have an inflamed appendix only. However, 4 (12.50%) had perforation at the shaft, and the base was healthy. All children with perforation had a diameter of > 12 mm and the presence of an appendicolith (Table 2).

Although there was no significant association between the duration of symptoms (p>0.05) and the presence of appendicolith (p>0.05) with the perforation rate, there was a substantial difference in the mean diameter of the appendix between patients with and without perforation (13.15 mm vs. 7.45 mm, P< 0.001).

There were no intra-operative complications related to LA, such as visceral injury, thermal burn, significant bleeding, conversion to open, and/or the need for an intra-abdominal drain.

Post-operatively, only 1 (3.13%) of patients developed superficial surgical site infection (Southampton Grade Ic) at the umbilical port through which the appendix was removed. His recovery was uneventful (Clavien-Dindo Grade I). However, there were no other postoperative complications, such as intra-abdominal abscess and/or need for readmission within 30 days.

No general anesthesia-related complications like cardiorespiratory events (hypoxemia, bradycardia, and hypotension) were recorded in this study.

The mean operating time for the surgery was 44.9 ± 15 minutes. This is the time from the skin incision for the first port to the closure of the last port (Table 3). The mean length of hospital stay was 2.59 ± 0.71 days, including the day of admission and the day of discharge. There was no significant association between the perforation rate and the length of hospital stay (p>0.05) (Table 3).

**Table 3:** Operative time and length of hospital stay

Characteristics		Frequency (n=32)	Percentage (%)	Mean + SD	‘P’ Value
Operating time (minutes)		-	-	44.9 ± 15	-
Length of Hospital Stay (LOS)	1-2 days	16	50.00	2.59 ± 0.71	0.32
	3-5 days	16	50.00		
Perforation		4	12.5	-	

## Discussion

As a minimally invasive technique, laparoscopy has added advantages in several areas, and many scholars have already proven this. Published comparative studies describe the benefits of LA over OA in terms of less postoperative pain, shorter hospital stays, better cosmetics, and faster return to regular activity [6,7]. The widespread use of LA is routinely recommended in hospitals where laparoscopic expertise and equipment are available [6].

Aligning with the common incidence at young and adolescent ages, more than 50% of the children in our study were between 10 and 15 years old, and the male predominance was also justified [2,3].

Common risk factors for appendicular perforation and complicated disease are extremes of age, duration since the onset of symptoms, the diameter of the appendix (>12 mm), and the presence of an appendicolith [2, 24, 25]. In a study by Samuel, the mean duration of symptoms was 55.2 + 26.4 hours [3]. The presentation duration among the children in our study was earlier (18 hours) than in other studies. The mean diameter was small (8.16 + 2.26 mm), and appendicolith was present only in 21% of cases. Aligning with the survey of Katipoglu et al., all four patients with a diameter >12 mm had perforation, and all perforated cases had appendicolith [25]. The early presentation of our children to the hospital could be the reason why we found most of the patients with uncomplicated acute appendicitis and a low rate of perforation.

Mechanical injury to the surrounding bowel and other visceral organs, thermal injury, port site and intra-abdominal bleeding, as well as conversion to open surgery, are some of the recorded complications of the laparoscopic approach. Other risks are intra-abdominal abscess, free air, feculent peritonitis, or, if advanced, septic shock [21, 26]. We had no such complications in any of our patients. This could be due to a small sample size, an experienced surgeon, no local inter-bowel adhesion or lump formation, etc. General anesthesia was also well tolerated during the procedure, similar to the available evidence [13,14].

Surgical site infection (SSI) has been reported in the range of 1.5 to 2.54% in children undergoing LA by various authors [21,27]. In our case, it was noted only in one patient (3.1%) and was superficial without further treatment. Post-operative intra-abdominal abscess (PIAA) is also reported in 0 to 5% of cases [10,11]. A study by Asarias et al. in the USA showed no significant difference in the occurrence of IAA after LA versus OA [28]. Our patient neither developed PIAA nor required readmission. All of this is due to the absence of postoperative complications and because of adequate intra-peritoneal lavage and drain under direct visualization of a laparoscope, which is limited in OA.

LA was always questioned regarding longer operating times. Operating time may be related to pediatric surgeons' and trainees' skills and experience. With increased knowledge, the mean operating time has become similar. Studies have shown operating times of 30-50 minutes for OA and 50-70 minutes for LA [10,11]. We had a similar result with a mean operating time of 44.9 + 15 minutes for LA. Operating time in our study was shorter than in the other studies (44 min vs. 78 min). Again, this may be due to the experience of our operating surgeon and early and uncomplicated cases.

Hospital stays following LA have always been shorter, as evident from previous studies [6,10]. In a study by Tsai et al. [10], the mean length of hospital stay in uncomplicated appendicitis was 2.4±1.1 days, similar to ours (2.59 + 0.71). It was slightly more than 4.1 ± 1.1 days in the case of perforated cases in the same study. It was even shorter, 1.7 ± 1.5 days, in the survey by Lee et al. [27].

Limitations

Our study is a single-centred, prospective study with a small sample size without a control group and proper randomization. We have not evaluated the cost factor for the surgery. Further large multi-centred prospective, randomized comparative study in Nepal with overall cost calculation is recommended.

## Conclusions

Acute appendicitis is the most common indication of surgical intervention in the pediatric age group. Laparoscopic appendectomy appears safe and feasible in the pediatric age group in the hands of experienced surgeons. Both intra-operative (visceral injury, thermal burn, bleeding, need of intra-abdominal drain, and conversion to open) and postoperative complications (SSI, PIAA, and readmission) are rare. This will further avoid a long and ugly abdominal scar, and the child can go home early. In the context of resource-limited countries like Nepal, and wherever laparoscopic equipment is available, laparoscopic appendectomy should be offered as a first choice for children presenting with acute appendicitis. Further large multi-centre similar studies with the ability to detect rare complications and overall cost are warranted in the context of Nepal to further support the above findings.
